# Family physicians can contribute to Olympic and Paralympic Games or other sports events

**DOI:** 10.1002/jgf2.507

**Published:** 2021-10-29

**Authors:** Ayano Hamai, Tadao Okada, Mamiko Ukai, Eiko Yoshizawa, Keita Kondo, Yuto Yamada, Mayu Kikuchi

**Affiliations:** ^1^ Department of General Medicine Awa Regional Medical Center Chiba Japan; ^2^ Tessyokai Kameda Family Clinic Tateyama Chiba Japan

## Abstract

Many sports physicians are from primary care backgrounds around the world but not in Japan. However, from the view of family physicians who contributed to Tokyo 2020 Olympic and Paralympic Games as medical staff, family physicians in Japan can play an active role in sporting events.

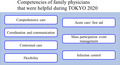

## CONFLICT OF INTEREST

The authors have stated explicitly that there are no conflicts of interest in connection with this article.

To the Editor,

Sports medicine is one of the fields that family medicine takes care of, and many sports physicians are from primary care backgrounds around the world but not in Japan. The team USA medical team included primary care physicians with an additional certificate qualification in sports medicine.^1^ The American Academy of Family Physicians contributed to the consensus statement for mass participation event management.^2^ As of October 2020, there are 6,420 Japan Sports Association‐certified sports doctors.^3^ However, only seven list their specialty as "general medicine." As a result of an extenuating circumstance leading to the addition of general medicine to the list in early 2021, many physicians may not have updated their status yet. Overall, few general physicians are involved in sports medicine in Japan.

We, the authors, are the attending physicians, fellows, and residents of a family medicine residency program in Japan. We participated in the TOKYO 2020 Olympic and Paralympic Games held in 2021 as on‐site medical staff.

Four of us contributed to the "polyclinic" at the athletes’ village, which handled the medical needs of the athletes and team staff. We were assigned to the internal medicine clinic and fever clinic, which were added to respond to the COVID‐19 situation. Other departments were orthopedics, dermatology, psychiatry, urology, ophthalmology, dentistry, female athlete clinic, physical therapy, and 24‐h emergency services. In past Olympics, the polyclinic included a department of general medicine or family medicine,^4^, ^5^ but not at TOKYO 2020. We took care of various problems beyond internal medicine category when the other departments were not staffed (such as otolaryngology and part‐time departments including dermatology and urology). As family physicians, wide and comprehensive scope of practice was helpful. We collaborated with the laboratory, radiology, and pharmacy departments.

At the fever clinic, we practiced nasopharyngeal swab polymerase chain reaction tests for patients with symptoms suspected of COVID‐19 or for patients who tested positive during screening saliva antigen tests (all athletes and staff, including us, were tested periodically). We worked with the logistics team and nurses and implemented careful infection control measures. It was essential to be flexible and cooperative in this unprecedented situation.

Four of us contributed to the venues, providing first aid for athletes and spectators. We participated in triathlon and fencing at the Olympics and para‐swimming, para‐triathlon, para‐fencing, sitting volleyball, and para‐taekwondo at the Paralympics. The venue medical team consisted of doctors, nurses, physical therapists, and first‐responders (not medical qualifiers). For the triathlon, special preparations for heatstroke were made. For sitting volleyball, care for trauma and chronic injuries, such as taping, was essential. For para‐swimming, responding to acute illnesses was critical. Although the system varied depending on the event, first aid and prehospital acute care were essential.

After TOKYO 2020, we reflected on the roles and competencies of family physicians. Table 1 shows the competencies that we found helpful during the event. Family medicine has a high affinity to sports medicine. Although family physicians need more training and exposure specific to sports medicine, family physicians in Japan should play an active role in sporting events.

**TABLE 1:** Competencies of family physicians that were helpful during TOKYO 2020

Competency	Detail
Comprehensive care	Handling a wide range of medical issues
Coordination and communication	Working with a multidisciplinary team Collaborating with physical therapists, especially on the field of play System‐based care with appropriate information sharing
Contextual care	Considering the diverse context of patients (athletes and team staff) from all over the world
Flexibility	Responding flexibly to the situation and changing systems
Acute care/ first aid	Implementing first aid and prehospital acute care on the field of play
Mass participation event management	Working as an organized team member Command and control, communication, and triage
Infection control	Implementing appropriate infection control measures

## References

[1] Nabhan D , Walden T , Street J , Linden H , Moreau B . Sports injury and illness epidemiology during the 2014 Youth Olympic Games: United States Olympic Team Surveillance. Br J Sports Med. 2016;50(11):688–93. 27098886 10.1136/bjsports-2015-095835

[2] Herring SA , Bergfeld JA , Boyajian‐O’Neill LA , Indelicato P , Jaffe R , Kibler WB , *et al* Mass participation event management for the team physician: a consensus statement. Med Sci Sports Exerc. 2004;36(11):2004–8. 15514519 10.1249/01.mss.0000145452.18404.f2

[3] Japan Sport Association [internet] Data on sports instructors. [updated Oct 2020 ; cited 20 Sep 2021]. Available from: https://www.japan‐sports.or.jp/coach/tabid248.html

[4] Vanhegan IS , Palmer‐Green D , Soligard T , Steffen K , O'Connor P , Bethapudi S , *et al* The London 2012 Summer Olympic Games: an analysis of usage of the Olympic Village ‘Polyclinic’ by competing athletes. Br J Sports Med. 2013;47(7):415–9. 23467963 10.1136/bjsports-2013-092325

[5] Kim D‐S , Lee Y‐H , Bae KS , Baek GH , Lee SY , Shim H , *et al* Pyeong Chang 2018 Winter Olympic Games and athletes’ usage of ‘polyclinic’ medical services. BMJ Open Sport Exerc Med. 2019;5(1):e000548. 10.1136/bmjsem-2019-000548 PMC6733333 31548900

